# Potential Biomarkers for Treatment Response to the BCL-2 Inhibitor Venetoclax: State of the Art and Future Directions

**DOI:** 10.3390/cancers13122974

**Published:** 2021-06-14

**Authors:** Haneen T. Salah, Courtney D. DiNardo, Marina Konopleva, Joseph D. Khoury

**Affiliations:** 1 College of Medicine, Alfaisal University, Riyadh 11533, Saudi Arabia; hsalah@alfaisal.edu; 2 Department of Leukemia, The University of Texas MD Anderson Cancer Center, Houston, TX 77030, USA; CDiNardo@mdanderson.org (C.D.D.); MKonople@mdanderson.org (M.K.); 3 Department of Hematopathology, The University of Texas MD Anderson Cancer Center, Houston, TX 77030, USA

**Keywords:** venetoclax, BCL-2, biomarkers, chemoresistance, treatment response

## Abstract

**Simple Summary:**

Apoptosis dysergulation is vital to oncogenesis. Efforts to mitigate this cancer hallmark have been ongoing for decades, focused mostly on inhibiting BCL-2, a key anti-apoptosis effector. The approval of venetoclax, a selective BCL-2 inhibitor, for clinical use has been a turning point in the field of oncology. While resulting in impressive improvement in objective outcomes, particularly for patients with chronic lymphocytic leukemia/small lymphocytic lymphoma and acute myeloid leukemia, the use of venetoclax has exposed a variety of resistance mechanisms to BCL-2 inhibition. As the field continues to move forward, improved understanding of such mechanisms and the potential biomarkers that could be harnessed to optimize patient selection for therapies that include venetoclax and next-generation BCL-2 inhibitors are gaining increased importance.

**Abstract:**

Intrinsic apoptotic pathway dysregulation plays an essential role in all cancers, particularly hematologic malignancies. This role has led to the development of multiple therapeutic agents targeting this pathway. Venetoclax is a selective BCL-2 inhibitor that has been approved for the treatment of chronic lymphoid leukemia and acute myeloid leukemia. Given the reported resistance to venetoclax, understanding the mechanisms of resistance and the potential biomarkers of response is crucial to ensure optimal drug usage and improved patient outcomes. Mechanisms of resistance to venetoclax include alterations involving the BH3-binding groove, *BCL2* gene mutations affecting venetoclax binding, and activation of alternative anti-apoptotic pathways. Moreover, various potential genetic biomarkers of venetoclax resistance have been proposed, including chromosome 17p deletion, trisomy 12, and *TP53* loss or mutation. This manuscript provides an overview of biomarkers that could predict treatment response to venetoclax.

## 1. Introduction

Hematologic malignancies frequently harbor dysregulation in type 1 programmed cell death, also known as apoptosis, resulting in a net pro-survival advantage [1]. Apoptotic cell death results from mitochondrial outer membrane permeabilization, which is tightly regulated through crosstalks between anti-apoptotic and pro-apoptotic proteins [2]. Perturbations in apoptosis control in hematologic malignancies result from overexpression of anti-apoptotic proteins and/or loss of pro-apoptotic proteins. Anti-apoptosis proteins share four conserved domains, referred to as B-cell lymphoma-2 (BCL-2) homology (BH) domains (BH1, BH2, BH3, and BH4), which play critical roles in the process of apoptosis [3]. 

Anti-apoptotic proteins, particularly BCL-2, are promising therapeutic targets in hematologic malignancies [4]. Indeed, Efforts to develop targeted BCL-2 therapy in hematologic malignancies have been longstanding [5]. Initial inhibitors were molecules that directly target the apoptotic pathway through dual BCL-2/BCL-X_L_ inhibition; these included ABT-737 (tool compound) and ABT-263 (navitoclax) [6]. Although navitoclax showed efficacy in preclinical trials, thrombocytopenia resulting from inhibition of BCL-X_L_, a megakaryocyte/platelet pro-survival factor, limited its clinical development [7]. The more selective BCL-2 inhibitor, ABT-199 (venetoclax), was developed subsequently and became the first BH3-mimetic approved by the United States Food and Drug Administration (FDA) for patients with chronic lymphocytic leukemia/small lymphocytic lymphoma (CLL) with chromosome 17p deletion. In patients with CLL/SLL, venetoclax is given orally as a daily monotherapy or in combination with other drugs [8]. More recently, venetoclax received FDA approval in combination therapy with azacitidine or decitabine or low-dose cytarabine to treat newly diagnosed adult acute myeloid leukemia (AML) patients unfit for standard intensive chemotherapy [9]. Although multiple clinical trials have demonstrated a favorable overall response rate to venetoclax [10,11], studies have identified mechanisms of resistance [12].

Biomarker studies can support therapy selection by identifying factors that impact sensitivity or resistance to a particular therapeutic option [13,14]. For example, biomarker identification can help predict which subpopulation of refractory CLL patients will benefit from treatment with venetoclax compared to alternatives such as ibrutinib, idelalisib, or duvelisib [13]. Several cytogenetic abnormalities, mutations, and alterations have also been identified as possible biomarkers of resistance or response to venetoclax therapy in myeloid malignancies. In this review, we discuss the molecular basis of the venetoclax mechanism of action and resistance, and we summarize current data and literature on molecular biomarkers associated with venetoclax response.

## 2. Molecular Basis of Venetoclax Activity

### 2.1. Apoptosis Activation and Control

Stimuli that trigger apoptosis may be extracellular (extrinsic) or intracellular (intrinsic). The extrinsic pathway is activated by the binding of ligands that activate surface cell death receptors, whereas the intrinsic pathway is dependent on internal cellular stress triggers such as DNA damage, growth factor deprivation, and hypoxia. Both the external and internal apoptosis pathways converge ultimately on caspase 3 and caspase 7, cysteine proteases that are the terminal effectors of apoptosis through cleavage of cellular proteins along aspartame residues.

Mitochondrial outer membrane permeabilization is a critical event in the progression of apoptosis; it results from the formation of pores that permit the release of cytochrome c into the cytoplasm [15]. Cytochrome c is a heme protein localized between the inner and outer mitochondrial membranes. When released into the cytoplasm as a result of mitochondrial membrane permeabilization, cytochrome c recruits procaspase 9 and apoptosis protease-activating factor 1, resulting in the formation of a catalytic multiprotein platform leading to caspase 9 activation and downstream cleavage of caspase 3. Mitochondrial outer membrane permeabilization also results in the production of second mitochondria-derived activator of caspase, which disengages X-linked inhibitor of apoptosis from caspase 3 leading to activation of the latter.

Mitochondrial outer membrane permeabilization and cytochrome c release are dynamically and intricately regulated by proteins whose net activation status allows the cell to oscillate between anti-apoptotic and pro-apoptotic states. Indeed, these BCL-2 superfamily proteins can be divided into three groups: anti-apoptotic BCL-2 family proteins, pro-apoptotic BH3-only proteins, and pro-apoptotic pore formers that include BAX (BCL-2-associated protein), BAK (BCL-2 homologous antagonist/killer), and BOK (BCL-2-related ovarian killer) [15,16]. Anti-apoptotic proteins localize to the outer mitochondrial membrane and prevent cytochrome c egress; they include BCL-2, BCL-X_L_, BCL-W, MCL-1, and A1. In healthy cells BCL-2 family proteins keep BAX and (Bcl-2 homologous antagonist/killer) BAK in check and inhibit their pore-forming ability. By contrast, BH3-only proteins such as BID (BHD interacting death domain) and BIM (BCL-2-interacting protein) localize to the cytoplasm and function upon ingress into the mitochondria by facilitating pore formation in the mitochondrial membrane or antagonizing BCL-2 and BCL-X_L_ by binding to them via BH3 domains.

All BCL-2 family proteins contain BH3, one of the four aforementioned conserved BH domains (BH1-BH4) that govern interactions between these proteins. Anti-apoptotic and pro-apoptotic pore-forming proteins contain all BH domains and have a highly conserved tertiary structure containing a hydrophobic BH3-binding groove. This structure is in contrast to that of pro-apoptotic BH3-only proteins, which lack other BH domains.

### 2.2. Apoptosis Dysregulation in Hematolymphoid Neoplasms

Evasion of apoptosis is recognized as a highly prevalent pathogenic feature in hematologic neoplasms, and it is a prominent feature of many B-cell and myeloid malignancies [2,17]. In view of the central role that BCL-2 proteins play in the intrinsic apoptosis pathway, there has been keen interest in taking aim at BCL-2 as a therapeutic target [4,18,19]. BCL-2 was initially identified as part of the t(14;18) chromosomal translocation in follicular lymphoma that results in upregulation of BCL-2 transcription and expression [20].

CLL is also characterized by a high level of BCL-2 expression, an essential factor for CLL cell survival [1,21]. Indeed, BH3 profiling (see Section 3.1.3) has demonstrated that CLL cells in most patients are dependent on BCL-2 for survival [21]. Furthermore, CLL cells commonly express high levels of BH3-only pro-apoptotic proteins, such as BIM, causing cells to be primed for cell death but dependent on BCL-2 function [1,22].

High rates of BCL-2 overexpression have also been reported in AML. For instance, a study found that almost 80% of AML cases overexpressed a pro-survival BCL-2 family protein [23]. Another study found BCL-2 overexpression in 87% of patients with newly diagnosed AML and in nearly 100% of relapsed patients [24].

Apoptosis dysregulation and BCL2 overexpression have been documented in a number of other hematolymphoid neoplasms [12,25]. A more detailed description is beyond the scope of this review. In the next sections, we focus on CLL and AML, as these are the major lymphoid and myeloid neoplasms, respectively, in which venetoclax-based therapies are used at present.

### 2.3. An Overview on BCL-2 Inhibition

BCL-2 family proteins bind to and inactivate BH3-containing proteins through a receptor-like groove that accommodates the ligand-like amphipathic α-helix BH3 motif. Multiple anti-BCL-2 agents have been developed over the past three decades and investigated in preclinical models and clinical trials. Broadly, they have included: (1) BH3 peptide mimetics; (2) mRNA-targeting antisense synthetic DNA oligonucleotides; (3) small-molecule BH3 mimetics.

BH3 peptide mimetics were developed to selectively bind anti-apoptotic BCL2-family proteins with high affinity, resulting in de-sequestration of pro-apoptotic proteins. Most agents in this category did not advance to clinical trials due to poor pharmacologic characteristics wherein agents with potent affinity had limited cellular permeability [26,27]. Among antisense oligonucleotides, oblimersen advanced into clinical trials and provided survival benefit in CLL [28] and multiple myeloma [29].

**Figure 1:**
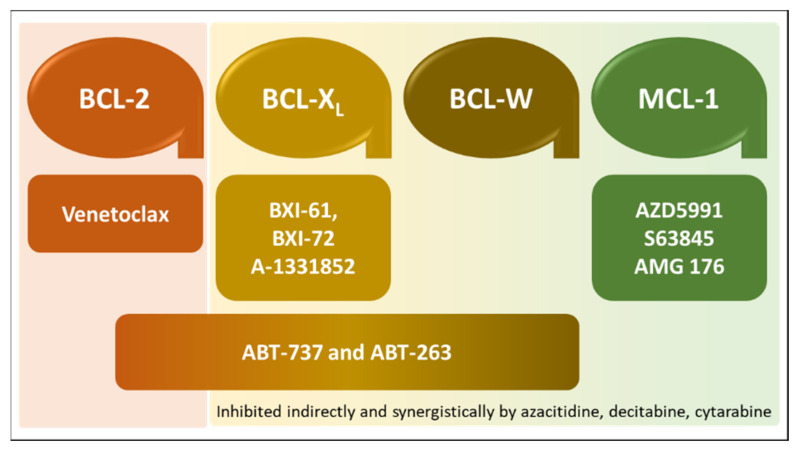
Small-molecule BH3-mimetic drugs in clinical use or under clinical evaluation. Inhibitors and their selectivity for anti-apoptotic BCL-2 family proteins are depicted with matching colors. ABT-737 and ABT-263 have broad selectivity, which spans BCL-2, BCL-X_L_, and BCL-W.

Small-molecule BH3 mimetics include natural and synthetic compounds capable of disrupting the interaction between anti-apoptotic BCL-2 family proteins and BH3-only proteins by competitive binding to the hydrophobic BH3-binding groove in the former [30,31]. Gossypol, a natural compound, and the synthetic compounds GX15-070 (obatoclax), ABT-263 (navitoclax), and ABT-199 (venetoclax) are such small-molecule inhibitors that have advanced into clinical trials [26]. Newer small-molecule BH3 mimetics in clinical use or under evaluation for clinical use and approval are summarized in Figure 1.

### 2.4. Venetoclax and Its Clinical Utility

Venetoclax is the first potent, selective BH3-mimetic BCL-2 inhibitor to be discovered. It will be the focus of the remainder of this review. Compared to navitoclax, venetoclax is highly selective for BCL-2, with >100-fold affinity in comparison to its affinity for BCL-X_L_ or BCL-W [32]. Exposure to venetoclax induces apoptosis potently in primary CLL cells, with downstream activation of caspase-9 and caspase-3 consistent with the activation of the intrinsic apoptotic pathway [33]. The first clinical testing of venetoclax was performed in patients with CLL refractory to standard therapies [11]. In a randomized, open-label, phase III trial, the combination of venetoclax plus rituximab resulted in significantly higher progression-free survival rates than bendamustine plus rituximab among patients with relapsed/refractory CLL/ [34].

In AML, the approval of venetoclax for use in combination therapy has resulted in a paradigm shift in leukemia treatment [9]. Presently, venetoclax is approved in combinations with azacitidine, decitabine, or low-dose cytarabine. Eligible groups include newly diagnosed adults 75 years or older and/or patients who have comorbidities that preclude the use of intensive induction chemotherapy. Early-phase trials showed that adding venetoclax to azacitidine improves AML remission rates [35]. In a pivotal phase III randomized, double-blind, placebo-controlled study, 431 untreated adult AML patients meeting at least one of the ineligibility criteria for intensive induction chemotherapy were included. Patients were randomized to receive azacitidine in combination with venetoclax or placebo. At a median follow-up of 20.5 months, the venetoclax plus azacitidine arm had longer overall survival and higher incidence of remission in comparison to the placebo arm [36].

Toxicities of venetoclax range from mild, such as diarrhea and nausea, to more significant side effects that include neutropenia-related infectious complications and tumor lysis syndrome. Thus, appropriate prophylactic measures, including tumor lysis syndrome prevention and antimicrobial prophylaxis, are recommended [36,37,38].

## 3. Mechanisms and Biomarkers of Resistance to Venetoclax

Malignant cells can find ways to evade death and bolster survival mechanisms as a result of baseline or therapy-induced selection (resistance) [38]. Many studies have identified intrinsic and acquired mechanisms of resistance to venetoclax [12,39]. Survival of cancer cells is determined by multiple anti-apoptotic proteins rather than a single anti-apoptotic protein [40]. Resistance mechanisms to venetoclax therapy fall under two main categories: (1) *BCL2* mutations impairing venetoclax binding; (2) activation of alternative anti-apoptosis pathways [41].

High levels of MCL-1 have been associated with venetoclax resistance in AML. For instance, the combination of venetoclax and MCL-1 inhibitor produced a synergistic apoptotic response in acute myeloid leukemia (AML) cells in vitro compared to the administration of venetoclax alone [42]. Additionally, Ramsey et al. [43] found that the use of venetoclax combined with VU661013, a selective MCL-1 inhibitor, reduced the expansion of AML cells and prevented MCL-1 resistance. In a pre-clinical study, Moujalled et al. [44] reported that co-targeting MCL-1 and BCL-2 was more effective against AML cells. In a study by Rahmani et al. [45], dual inhibition of PI3K/mTOR pathway and BCL-2 showed significant MCL-1 downregulation in AML cells in vitro and in vivo. Another study has found that combining CDK-selective inhibitor (voruciclib), potently blocks CDK-9 (the transcription regulator of MCL-1) and enhances venetoclax action by inducing tumor cell apoptosis [46]. Table 1 summarizes different reported mechanisms of resistance to venetoclax.

The variety of possible resistance mechanisms to venetoclax are underscored by the fact that the complete remission rate for venetoclax monotherapy is relatively low [45,47]. Hence, other mechanisms to improve the efficacy of venetoclax are needed [45,48].

With the development of genomic profiling techniques and selective molecular targeted therapies, the role of biomarkers to predict treatment effect or native/emergent resistance has gained increased importance in cancer treatment [14,49]. In the context of venetoclax therapy, BCL-2 protein expression alone does not predict response, and additional biomarkers pertinent to anti- and pro-apoptotic protein families are warranted [50]. The following section describes the mechanisms and biomarker of venetoclax response primarily in CLL and AML.

### 3.1. Mechanisms and Biomarkers of Venetoclax Response in CLL

#### 3.1.1. BCL2 Mutations of Venetoclax Binding Site

During the past few years, one of the most significant developments in understanding venetoclax resistance included the identification of *BCL2* mutations that impact adversely venetoclax binding affinity. Blombery et al. [51] analyzed paired pre-venetoclax and progression samples of 15 patients with CLL. Patients had serial samples available throughout the study. The *BCL2*p.Gly101Val mutation was identified in 7 patients who developed resistance after long-term treatment ranging from 19 to 42 months after being initially responsive. The median time on venetoclax to clinical progression was about 36 months. This mutation was not present before therapy initiation, and it reduced the affinity of venetoclax binding to BCL-2 by almost 180-fold. It involves a highly conserved residue that in tertiary configuration faces the venetoclax-binding groove. Birkinshaw et al. [55] determined the crystal structure of venetoclax when bound to wild-type BCL-2 and the mutant BCL-2 Gly101Val. Another study conducted on seven venetoclax-resistant leukemia and lymphoma cell lines revealed resistance mediated by mutations involving residue 104, whereby phenylalanine is replaced by either leucine (Phe104Leu) or cysteine (Phe104Cys) impacting the BH3-binding groove [52]. Other mutations included Asp103Tyr/Glu/Val, Val156Asp, Arg107_Arg110dup, Ala113Gly, and Arg129Leu. Of note, these mutations were not detectable before the exposure of venetoclax therapy [41,56]. A study by Tausch et al. [57] identified the mutant D103Y using next-generation sequencing in patients refractory to venetoclax therapy.

#### 3.1.2. Activation of Alternative Anti-Apoptotic Proteins and Pathways

As venetoclax is highly selective for BCL2, selective pressure leading to activation of other BCL-2 family members—particularly MCL-1—has been identified as a major mechanism of resistance [54,58,59,60]. As it is, amplification of the *MCL1* gene locus on chromosome 1q21.2 is highly prevalent in human cancers and correlates with MCL-1 dependency [61]. Thijssen et al. [53] identified increased expression of BCL-X_L_, MCL-1, and BFL-1 in lymph nodes of CLL patients resistant to venetoclax. In one study, three out of six CLL patients who had venetoclax-resistant progressive disease had chromosome 1q amplification [54]. Oppermann et al. [47] suggested that cells may develop kinase-mediated survival signals in their proliferation centers (lymphoid tissue and bone marrow), resulting in upregulation of anti-apoptotic signals such as BCL-X_L_, MCL-1, and A1. The study suggested that sunitinib, a clinically available kinase inhibitor, augmented the cellular killing of venetoclax [47]. Song et al. [62] suggested along similar lines that a structural change in the BH3-binding groove is caused by phosphorylation of BCL-2, providing a basis to overcome this resistance of CLL cells by adding kinase inhibitors with venetoclax therapy.

Other mechanisms in this category include cellular alterations that lead to the reduction or loss of negative regulators of BCL2. For instance, del(13q14) leads to loss of miR-15 and miR-16, both negative posttranscriptional regulators of BCL-2, in CLL [54,63].

#### 3.1.3. BH3 Profiling as a BH3-Mimetic Drug Response Prediction Tool

BH3 profiling is a peptide-based technique used to predict sensitivity to cancer drugs by measuring the ability of different BH3 peptides to induce mitochondrial depolarization [64,65]. BH3 profiling determines the functional dependence of a cell on specific anti-apoptotic proteins for survival [66,67]. During BH3 profiling, target cells are permeabilized by digitonin then exposed BH3 peptides from various BCL-2 family proteins. Cytochrome c release and mitochondrial outer membrane permeabilization are then measured using various detection platforms. This process determines the likelihood of cells to undergo apoptosis—priming for apoptosis—and the particular anti-apoptotic protein(s) that the cells are dependent on for survival [64]. BH3 profiling can be used to determine potential sensitivity to drugs such as venetoclax [21]. In CLL cells, BH3 profiling revealed that CLL cells from peripheral blood are highly primed and such priming is associated with improved clinical response [68]. BH3 profiling can potentially provide a fast and affordable way to assess sensitivity to venetoclax and predict anti-apoptotic proteins dependence [64,65,69]. Certo et al. [66] illustrated that BH3 profiling can differentiate between MCL-1 cellular dependence and BCL-2 cellular dependence, both of which have been increasingly recognized as individual potential biomarkers to venetoclax. One of the limitations of BH3 profiling includes difficulty in determining optimal peptide concentrations [64].

One study developed a screening strategy to determine which tumors fail to respond to BH3 mimetics by exposing different cell lines to different combinations of venetoclax, a selective BCL-X_L_ inhibitor, and a selective MCL-1 inhibitor [70]. The level of BCL-2 was found to predict sensitivity to monotherapy; additionally, BFL-1 and BCL-W activation promoted resistance in all tested combinations of BCL-2, BCL-X_L_, and MCL-1 inhibitors. Guièze et al [54]. performed gain-of-function genetic modifier screens and showed that MCL-1 and BCL-2 family (mainly BCL-2, BCL2L1, and BCL2L2) protein upregulation resulted in venetoclax resistance in CLL cells. These proteins were recommended as possible biomarkers to venetoclax therapy.

#### 3.1.4. Mutational Alterations Correlating with Venetoclax Response

Potential disease-specific biomarkers for patients with hematological malignancies treated with venetoclax have been proposed. Roberts et al. [71] have identified three categories of factors that correlate with shorter duration of response to venetoclax in CLL; they include: 1) bulky disease; 2) refractoriness to fludarabine or B-cell receptor pathway inhibitors (BCRi); 3) an adverse mutation profile (i.e., *TP53* loss or mutation, *NOTCH1* mutation, and *IGHV* unmutated status). These were identified as essential biological markers of disease with a propensity to progress with ongoing BCL-2 inhibition. In patients with relapsed/refractory CLL, resistance to venetoclax could be approached by the timing of when the resistance develops during therapy. In early progressor patients, associated genomic changes include karyotypic complexity, loss of *CDKN2A/B*, *BTG1* mutation, and *NOTCH1* mutation [72]. These findings were reported in other studies showing that in patients with relapsed or refractory CLL on oral venetoclax monotherapy, both *NOTCH1* and *SF3B1* mutations correlate with shortened duration of response [71,73].

#### 3.1.5. Cytogenetic Alterations Correlating with Venetoclax Response

Chromosome 11q deletion, del(11q), has been associated with progressive CLL [74]. In patients with relapsed or refractory disease that have del(11q), it was found that treatment with venetoclax monotherapy is not correlated with improvement or complete response (CR) [71].

Trisomy 12 is the third most common chromosomal abnormality in CLL (10-20%) [74]. Trisomy 12 has been considered an intermediate or low risk prognostic factor [74]. Trisomy 12 is considered an early event in CLL evolution that enables the emergence of alterations in genes such as *NOTCH1*, *TP53*, and *FBXW7* [74]. In patients with relapsed or refractory CLL on oral venetoclax monotherapy, trisomy 12 was not correlated with the likelihood of achieving a CR [71].

Chromosome 13q deletion, del(13q), which is found in more than 50% of CLL, is the most common cytogenetic abnormality detected by FISH. It has been historically associated with a favorable prognosis. In relapsed or refractory CLL patients treated with venetoclax, it has been shown that del(13q) is associated with an improved CR [71].

Although *TP53* abnormalities are uncommon at diagnosis (5–10%), they are found in 40–50% of therapy-refractory or advanced CLL cases [75]. Deletion of 17p is found in 3–8% of CLL patients at diagnosis; however, this frequency increases to 30% in patients with refractory disease. Patients with del(17p) have always been considered in the highest risk prognostic category, with the shortest OS and PFS [74]. Different studies have confirmed that response to venetoclax is independent of p53 functional status [1,71].

### 3.2. Mechanisms and Biomarkers of Venetoclax Response in AML

#### 3.2.1. *BCL2* Gene Alterations

*BCL2* mutations involving the venetoclax-binding site do not constitute a common pathway of resistance in AML. This has been postulated to be due to the typically short duration of venetoclax therapy in AML (in comparison to CLL) and/or to the sensitivity of AML cells with mutant *BCL2* to combination therapy [76].

#### 3.2.2. Mutational Alterations Correlating with Venetoclax Response and Activation of Alternative Anti-Apoptosis Pathways

*FLT3* internal tandem duplication (ITD) mutation and *PTPN11* mutations are associated with primary or acquired resistance to venetoclax monotherapy. In addition, upregulation of the Ras/MAPK pathway is an important factor in determining venetoclax resistance [77]. Collectively, these pathogenic alterations result in MCL-1 upregulation, circumvented by targeted MCL-1 inhibition [78]. Indeed, the interplay between different anti-apoptotic proteins has led to multiple studies exploring synergy in targeting BCL-2 and MCL-1 using combination drugs [43,44]. Hormi et al. [79] reported that the use of a specific MCL-1 inhibitor (S63845) in combination with venetoclax induced apoptosis in AML cells, and such a response was also detected in venetoclax-resistant AML cells. Additionally, AML cell lines with *MLL* fusion genes were more sensitive to venetoclax therapy and that the BCL-2/MCL-1 ratio represents a useful biomarker for predicting response to venetoclax [80].

Other mutations have been also suggested to impact response to venetoclax. The impact of *IDH1* and *IDH2* mutation status on the sensitivity to venetoclax as a single agent is significant, and such patients appear to benefit preferentially from venetoclax therapy [42]. In patients with secondary/refectory AML treated with venetoclax monotherapy, those with *SRSF2* or *ZRSR2* mutation had a 70% reduction in bone marrow blasts. In comparison, 21% of patients with *FLT3*-ITD mutation did not have a decrease in bone marrow blasts, and 29% of patients with *PTPN11* mutation did not have a decrease in bone marrow blasts [81]. Additionally, AML cell lines with overexpression of VMP1 (an autophagy protein) have increased autophagy, which increased the threshold for mitochondrial outer membrane permeabilization, mediating venetoclax resistance [82].

The prognostic importance of *IDH1/2* mutations is influenced by co-mutational status and the specific locations of the mutations [83]. In vitro and clinical observation have suggested increased sensitivity of *IDH1/2*-mutant AML cells to pharmacologic BCL-2 inhibition induced by (R)-2-hydroxyglutarate-mediated inhibition of cytochrome c oxidase activity leading to lowering of the mitochondrial threshold to trigger apoptosis [83,84,85]. In patients with secondary/refractory AML on venetoclax monotherapy, *IDH1/2* mutations were associated with an improved PFS [86]. This was further supported in a recent phase III clinical trial comparing combination of therapy with venetoclax and azacitidine with azacitidine and placebo in AML. Subgroup analysis based on *IDH1/2* mutation status showed higher OS in the combination therapy group [36] Acquired biallelic silencing of *TP53* correlated with initial responsiveness followed by refractoriness in older patients with AML [87].

## 4. Conclusions

In this review, we discussed BCL-2 inhibitors and highlighted their biology, mechanism of action, and emerging clinical impact. Notably, we highlighted emerging patterns of resistance to anti-BCL-2 inhibitors with primary emphasis on venetoclax. Up to date, there is no sufficient clinical data for treating patients who develop venetoclax resistance. In CLL, potential therapeutic options are available for patients who develop venetoclax resistance, such as BTKi, PI3Ki, and CAR-T cell therapy. Interestingly, novel BH3 mimetics are being studied as a potential alternative to venetoclax. Furthermore, considerable progress has been made in finding MCL-1 inhibitors. Up to date, 3 MCL-1 inhibitors have been used in pre-clinical studies and might show promising results in overcoming venetoclax resistance. As mentioned earlier, combining the CDK-selective inhibitor (voruciclib) with venetoclax is a possible way to overcome resistance, as it indirectly lowers MCL-1 transcription. BH3 profiling could also be a potential assay that leads to the personalization of therapy even before treatment initiation, as it measures dependencies on anti-apoptotic proteins. In this review, we tried to highlight potential biomarkers to venetoclax resistance. However, further work and clinical trials are needed. As these therapies establish their place among treatment options for various hematolymphoid neoplasms, continued understanding of predictive biomarkers of treatment response to anti-BCL-2 inhibitors gain further importance to personalize treatment and predict treatment response.

## Figures and Tables

**Table 1:** Summary of resistance mechanisms to venetoclax therapy.

Pathways of Resistance	Reported Examples of Mechanisms of Resistance	Type of Malignancy	Type of Study	Reference
*BCL2* mutations of venetoclax-binding site	Gly101Val mutation *	CLL	Patients derived samples	[51]
	Phe104Leu/Cys mutations	DLBCL, FL, MCL, and leukemia cell lines	In vitro (preclinical)	[52]
Alternative anti-apoptotic proteins pathways	Overexpression of BCL-X_L_, MCL-1, and BFL-1.	CLL	Patients derived samples	[53]
	Amp1q leading to MCL-1 overexpression	CLL	In vitro (preclinical) followed by patients derived samples	[54]
	Kinase-mediated survival signals leading to BCL-X_L_, MCL-1, and A1	CLL	Patients derived samples	[47]
	MCL-1 overexpression	AML	In vitro (preclinical)	[42]
	MCL-1 overexpression	AML	In vitro (preclinical)	[43]
	PI3K/mTOR pathway and BCL-2 → MCL-1 overexpression	AML	In vitro (preclinical)	[45]
	Cyclin-dependent kinase 9 inhibition (CDK-9) → MCL-1 overexpression	DLBCL	In vitro (preclinical)	[46]

Abbreviations: CLL: chronic lymphoid leukemia, DLBCL: diffuse large B-cell lymphoma, FL: follicular lymphoma, MCL: mantle cell lymphoma, and AML: acute myeloid leukemia. * Resistance 19–42 months after being initially responsive to venetoclax.
